# Programming With Varying Dietary Fat Content Alters Cardiac Insulin Receptor, Glut4 and FoxO1 Immunoreactivity in Neonatal Rats, Whereas High Fat Programming Alters Cebpa Gene Expression in Neonatal Female Rats

**DOI:** 10.3389/fendo.2021.772095

**Published:** 2022-01-05

**Authors:** Annelene Govindsamy, Samira Ghoor, Marlon E. Cerf

**Affiliations:** ^1^Discipline of Pharmaceutical Sciences, University of KwaZulu-Natal, Durban, South Africa; ^2^Biomedical Research and Innovation Platform, South African Medical Research Council, Cape Town, South Africa; ^3^Grants, Innovation and Product Development, South African Medical Research Council, Cape Town, South Africa

**Keywords:** diabetes, fetal programming, insulin resistance, insulin signaling, metabolic syndrome, nutrition, obesity

## Abstract

Fetal programming refers to an intrauterine stimulus or insult that shapes growth, development and health outcomes. Dependent on the quality and quantity, dietary fats can be beneficial or detrimental for the growth of the fetus and can alter insulin signaling by regulating the expression of key factors. The effects of varying dietary fat content on the expression profiles of factors in the neonatal female and male rat heart were investigated and analyzed in control (10% fat), 20F (20% fat), 30F (30% fat) and 40F (40% fat which was a high fat diet used to induce high fat programming) neonatal rats. The whole neonatal heart was immunostained for insulin receptor, glucose transporter 4 (Glut4) and forkhead box protein 1 (FoxO1), followed by image analysis. The expression of 84 genes, commonly associated with the insulin signaling pathway, were then examined in 40F female and 40F male offspring. Maintenance on diets, varying in fat content during fetal life, altered the expression of cardiac factors, with changes induced from 20% fat in female neonates, but from 30% fat in male neonates. Further, CCAAT/enhancer-binding protein alpha (*Cebpa*) was upregulated in 40F female neonates. There was, however, differential expression of several insulin signaling genes in 40F (high fat programmed) offspring, with some tending to significance but most differences were in fold changes (≥1.5 fold). The increased immunoreactivity for insulin receptor, Glut4 and FoxO1 in 20F female and 30F male neonatal rats may reflect a compensatory response to programming to maintain cardiac physiology. *Cebpa* was upregulated in female offspring maintained on a high fat diet, with fold increases in other insulin signaling genes viz. *Aebp1*, *Cfd* (adipsin), *Adra1d*, *Prkcg*, *Igfbp*, *Retn* (resistin) and *Ucp1*. In female offspring maintained on a high fat diet, increased *Cebpa* gene expression (concomitant with fold increases in other insulin signaling genes) may reflect cardiac stress and an adaptative response to cardiac inflammation, stress and/or injury, after high fat programming. Diet and the sex are determinants of cardiac physiology and pathophysiology, reflecting divergent mechanisms that are sex-specific.

## Introduction

Fetal programming is defined as the predisposition of the fetus to metabolic abnormalities due to stimuli or insults during critical developmental phases. The fetus is highly responsive to any alterations within the placental environment, and its nutritional status is an integral component that affects both growth and maturation (1). Insulin resistance, which is closely associated with obesity, predisposes to cardiovascular disease, diabetes and metabolic syndrome (2, 3) – pathologies that contribute greatly to global morbidity and mortality (4). However, independent of overt diabetes, altered glucose homeostasis impacts the autonomic functions of the heart, culminating in an increased probability for developing cardiac disease (5). Diet is a major contributor to multiple metabolic conditions, where high fat diets (HFDs), rich in trans and saturated fatty acids, correlate to insulin resistance (6, 7). A HFD can be used to induce programming effects in offspring i.e. high fat programming. Since type 2 diabetes and obesity are closely associated, most diabetes models display an obese phenotype (8). A maternal HFD and/or maternal gestational obesity contribute to the origin of metabolic disease in the offspring, are associated with congenital abnormalities, and may increase neonatal morbidity and mortality (9) with gestational HFDs adversely affecting the offspring’s metabolic physiology (10). Maternal gestational obesity predisposes offspring to diabetes, insulin resistance and hyperinsulinemia (11, 12). Rats maintained on a HFD during pregnancy and lactation were insulin resistant and glucose intolerant (13), with a long-term maternal HFD suggested to induce maternal insulin resistance and alter offspring neuroendocrine systems (14). In normal pregnancies, hyperlipidemia presents; in diabetic pregnancies, hyperlipidemia is exacerbated (15) and fetuses are exposed to hyperglycemia. In diabetic pregnancies, the compromised maternal metabolic milieu therefore exposes the developing fetus to elevated glucose and lipids, that stimulate fetal hyperinsulinemia and adversely affect the developing fetal heart (16). In offspring from mothers with diabetic pregnancies, maintenance on a HFD further impaired diastolic and systolic function through oxidative stress, mitochondrial dysfunction, the accumulation of lipid droplets and metabolic derangements (16).

In obesity, cardiac insulin resistance is an early adaptive event, which develops prior to insulin resistance in other organs, and can be induced in rodents after 10 days of high fat feeding (17). After maternal overfeeding (HFD + high sugar diet), fetal hearts exhibited dysregulated pathways e.g. the overactivation of the c-Jun N-terminal kinase (JNK)-insulin receptor substrate 1 (IRS1) pathway and AMP-activated protein kinase (AMPK, a cardioprotective factor) downregulation (18). Mouse offspring from obese mothers were hyperinsulinemic with enhanced cardiac insulin signaling evident by upregulation of the distal insulin signaling pathway viz. p-AKT (or protein kinase B), p-ERK (extracellular signal-regulated kinase), p-mTOR (mammalian target of rapamycin) and p38-MAPK (mitogen-activated kinase) (11). Cardiac insulin resistance impairs cardiac metabolic efficiency and can induce contractile dysfunction (19, 20).

Approximately 60%-80% of cardiac energy requirements are met by fatty acid metabolism (21). Abnormalities in lipid uptake or intracellular metabolic activities may be causal in the etiology of heart disease, excluding dilated cardiomyopathy due to metabolic aberrations (22). Lipotoxic cardiomyopathy (or fatty heart) is characterized by cardiac dysfunction attributed to excess lipid accumulation (23). Lipid droplets are typically present in the hearts of diabetic and metabolic syndrome patients (24–26). With multiple gaps in the knowledge, and whilst the consequential effect of diet on ventricular hypertrophy and cardiac function is somewhat controversial, left ventricular hypertrophy was reduced in hypertensive rodents fed a 60% fat diet concomitant with dysfunctional systole (27). Fatty acid oxidation was elevated in rodents fed a HFD compared to rodents on a diet low in fat and rich in carbohydrate content (28).

There are several key factors required for maintaining cardiac integrity (i.e. cardiac physiology and structure). Insulin receptors are tyrosine kinase derived transmembrane receptors (29). Defects in their structure and subsequent activity are a significant area of current research; however, cardiac insulin resistance has not been extensively investigated (30). Glucose transporter 4 (Glut4) is a major contributor in the uptake and removal of glucose from the circulation, and consequently a salient regulator of systemic glucose homeostasis (31). Glut4 belongs to a family of 13 transporter proteins (Glut1-12 and H^+^/myoinositol co-transporter (HMIT)) (32) that facilitate sugar-substrate translocation and are encoded in the mammalian genome (33, 34). Glucose transporters are highly expressed during postnatal development and in the adult heart (35). The transcription factor, forkhead box protein 1 (FoxO1), displays multiple functions in the regulation of apoptosis, senescence, proliferation, stress resistance, metabolism, differentiation and autophagy (36, 37). Therapy aimed at cardiac-derived FoxO1 could reduce mortality caused by heart failure in diabetic patients (38). *Cebpa* and *Cebpb* play roles in the onset of abdominal obesity, and are linked to altered adipokine levels, cardiovascular disease and diabetes (39). The *Cebpb* gene resides on chromosome 20q13.1 (40), with linkages to traits for diabetes, obesity and insulin (41–47).

Optimal cardiac development and physiology requires balanced and adequate intrauterine nutrition (macro- and micronutrients), with sufficient oxygen to the fetus, to enable offspring to manage metabolic stressors that provoke the pathogenesis of cardiovascular disease (48). Different sex mechanisms of fetal cardiac programming can be induced by a suboptimal *in utero* environment (49) e.g. placental insufficiency, hypoxia, protein deficiency or high fat programming. Genetic, epigenetic and hormonal factors contribute to sex specificity (50). In offspring, high fat programming induces sex-specific metabolic alterations (51), such as sex-specific hepatic fat accumulation (52) and altered histone binding at the leptin receptor (Lepr) promoter in female offspring’s hippocampus and interleukin-6 (IL-6) with epigenetic deregulation of Lepr in female hippocampal neurons (*in vitro*) (53). In the murine placenta, maternal diets shape gene expression with female placentae more sensitive to nutritional programming; nutrition has a global effect on gene expression that is specific, and female fetuses respond more robustly, with distinct sexual dimorphic gene expression (54).

Surprisingly, few studies on cardiac lipid metabolism have been conducted relative to adipose tissue and liver metabolism studies (22). In our previous studies, using diets varying in fat content, most (~90%) of the circulating fatty acids that were analyzed in neonates were unaltered (55). However, neonates maintained on 20% and 30% fat diets (as energy) *in utero* (i.e. 20F and 30F neonates respectively), displayed increased crown to rump length, higher body weights (56), with reduced heart weights in 30F and 40F neonates (40F neonates were maintained on a 40% fat diet *in utero*), elevated glycemia in the 20F neonates, and no differences in insulinemia and glucagonemia (55). Litter size and maternal body weight did not play a role in 40F mothers, but the caveat was that mothers maintained on a HFD throughout gestation had reduced food intake compared to the control and 20F mothers, that may have contributed to their offspring’s stunted growth and development (55). Studies have examined the programming effects of a HFD on insulin signaling in the liver and muscle, however minimal information is available on fetal cardiac insulin signaling. Insulin signaling and resistance are implicated in the pathogenesis of cardiovascular disease. The targeting of IRS1 and IRS2 through the activation of the Akt [Protein kinase B (PKB)] and FoxO1 signaling cascade and the related protein kinases and target genes is critical for diabetes prevention and treatment and the accompanying cardiac dysfunction (57). To determine the sexual dimorphic effects, we therefore investigated, in female and male neonatal offspring, the effect of (i) maternal diets of 20%, 30% and 40% (40% fat which is a HFD to induce high fat programming) fat on cardiac immunoreactivity for insulin receptor, Glut4 and FoxO1; and (ii) a maternal gestational HFD on cardiac insulin signaling gene expression.

## Methods

### Study Design

Following institutional ethical approval, three-month old virgin Wistar rats weighing 220-275g were paired for mating (55). Upon confirmation of pregnancy, dams were removed, individually housed and categorized into groups (n = 4 per group). Pregnant rats were fed diets (all patties) of 10% (control), 20% (20F), 30% (30F) and 40% (HFD) fat as energy throughout gestation, with free access to water. The dietary macronutrient profiles were Control [10.69% fat, 15.13% protein, 74.16% carbohydrates with 453.37 total kcal/100g]; 20F [20.68% fat, 15.09% protein, 64.22 carbohydrates with 525.51 total kcal/100g]; 30F [31.00% fat, 15.77% protein, 53.23% carbohydrates with 554.08 total kcal/100g] and 40F [40.17% fat, 15.09% protein, 44.73% carbohydrates with 600.81 total kcal/100g] (55). Protein was constant at 15% in all the diets (to avoid the effects of protein deficiency); the fat comprised saturated fatty acids (viz. myristic, palmitic and stearic acid) and the mono-unsaturated fatty acid, oleic acid, derived from animal fat; with carbohydrates mainly derived from starch to mimic a westernized diet (58). In each one-day old rat, after euthanasia, hearts were harvested and either snap frozen in liquid nitrogen or processed through paraffin-wax fixation (55). Offspring were randomly assigned, from each dam, to ensure that each dam accounted for an even distribution of offspring across all the groups. There were no differences in litter sizes, gender distribution, circulating total triglyceride and total free fatty acid concentrations (55).

### Immunohistochemistry and Image Analysis

Heart tissue embedded in paraffin wax (4 μm sections) were set on microscopic slides and de-waxed (twice) in xylene at room temperature (10 minutes each). Tissue sections were then rehydrated in the following solutions at room temperature: 100% ethanol twice (5 minutes each), absolute methanol for 20 minutes, 90% ethanol for 5 minutes, 70% ethanol for 5 minutes, 50% ethanol for 5 minutes and deionized in distilled water for 5 minutes. Slides were placed in 0.1 M sodium citrate (pH 6.0) and boiled for 5 minutes at 80°C. Thereafter, slides were cooled to room temperature for 30 minutes and immersed in deionized distilled water for 5 minutes. Slides were placed in 20% hydrogen peroxide (in methanol) for 20 minutes and repeated four times in total. Following this inhibition of endogenous peroxidase activity, slides were washed twice in tris-buffered saline (TBS) for 5 minutes each. TBS/BSA (tris-buffered saline/3% bovine serum albumin/10% casein buffer which is a blocking agent) was added to each slide (100 μl) for 1 hour (fresh blocking agent applied every 30 minutes). Finally, antibody [anti-insulin receptor (C-terminal; Sigma, USA), 1:50; anti-Glut4 (Abcam, UK), 1:500; anti-FoxO1 (Abcam, UK), 1:75] was added to individual slides and incubated in a humidified chamber for 18 hours at 4°C.

Following incubation, the slides were allowed to come to room temperature and a secondary antibody (Vector Lab, USA) was applied for 10 minutes followed by the ABC Elite kit (Vector Labs, USA) as per manufacturer’s instructions. The chromogenic agent DAB (3,3’-Diaminobenzidine) was added to the slides for up to 5 minutes, and the reaction was quenched with TBS. Tissues were counterstained in Mayer’s hematoxylin for up to 5 minutes and washed under running water. Slides were then dehydrated i.e. the reverse of rehydration from deionized distilled water to 100% ethanol, prior to placing them in xylene for approximately 5 minutes. Finally, slides were mounted in a permanent medium (DPX, Merck, South Africa) and viewed under light microscopy.

Immunostained slides for insulin receptor (combined offspring: n = 8-10; female offspring: n = 3-6; and male offspring: n = 4-7), Glut4 (combined offspring: n = 9-12; female offspring: n = 3-6; and male offspring: n = 5-7) and FoxO1 (combined offspring: n = 8-10; female offspring: n = 3-6; and male offspring: n = 5-7) were examined by employing a bright field/phase contrast microscope (DMLB; Leica, Germany) attached to a camera (DFC 300FX, Leica). The adaptor magnification was X10 and the objective lenses (Leica) used X20, X40 and X100 magnification which had numerical apertures of 0.50, 0.70 and 1.3, respectively. Tagged image format (24-bit TIFF) images were captured (Leica LAS image capturing software). Immunostaining was quantified by converting the archived 24-bit TIFF images to 8-bit binary images with a grey scale of 256 phases. Specific immunostained grey scale intensities were quantified by image analysis (AnalySIS Five, Soft Imaging Systems, Germany) with pre-determined threshold range-limited pixel/μm^2^ values. Any non-specific background immunostaining, calculated from staining in method controls, was discounted from all positive immunostaining.

### Enzyme-Linked Immunosorbent Assay (ELISA)

Frozen neonatal hearts were thawed on ice followed by homogenization on ice in 1 ml of PBS (Dounce Homogenizer). Samples were centrifuged (Eppendorf, Germany) at 5000 x g for 5 minutes to retrieve the supernatant and diluted 1:1 in PBS (5 μl supernatant: 5 ml PBS) for ELISA (Rat Glut4 ELISA Kit, Elabscience, USA) analysis.

In a 96-well ELISA plate, 100 μl of the standard working solution (40 ng/ml), constituted from the reference standard, and sample diluent was added to the first two columns. A volume of 100 μl of the sample was added to the remaining wells, in triplicate. The plate was incubated (in a water bath) at 37°C for 90 minutes. Following the removal of all liquid from the plate, 100 μl of biotinylated detection antibody was added and the plate was incubated at 37°C for 60 minutes. The wells were aspirated, washed thrice (with wash buffer), 100 μl of horse radish peroxidase conjugate was added, followed by incubation at 37°C for 30 minutes. The liquid was aspirated from the wells, the plate was washed five times, and 90 μl of substrate reagent was added to each well, followed by incubation at 37°C for 15 minutes. The reaction was stopped by adding 50 μl of stop solution to each well. All reagents used in the assay were provided in the ELISA kit. The Glut4 concentrations (combined offspring: n = 3-6; female offspring: n = 3; male offspring: n = 3 for control, 30F and 40F but n = 0 for 20F) were estimated by the mean measurements at 450 nm using an ELISA plate reader (microplate reader, BioRad 3550, BioRad, UK) by interpolation from the standard curve.

### RNA Isolation and mRNA Gene Expression

Heart tissue (~20 mg), was harvested from male (n = 10) and female (n = 10) neonatal rats, immersed in 1 ml of RNA later (Ambion, Invitrogen, Thermofisher Scientific, USA) and stored at -80°C. Total RNA was isolated with the RNeasy mini kit (Qiagen, Germany). Briefly, in a 2 ml Eppendorf tube, the heart tissue (11-20 mg) was homogenized in 600 μl RLT buffer (with 10 μl β-mercaptoethanol per 1ml of RLT buffer) and a stainless-steel bead using the Qiagen tissue lyser. The lysate was centrifuged at 4°C and the supernatant was transferred to a new 2 ml tube. Then 70% ethanol was added to the supernatant, mixed and centrifuged at 15000 rcf. Half of this volume was added to a spin column provided in the kit and centrifuged at 15000 rcf for 15 seconds. The flow-through was discarded, and the step was repeated with the remainder of the supernatant/ethanol mixture. Subsequent steps where buffers RW1 and RPE were added to the spin columns were followed up and included the elution of RNA with 50 µl of RNase free water. The isolated RNA was treated with Dnase using TURBO-DNA free (Ambion) to remove any gDNA contamination. The nanodrop spectrophotometer was used to quantify and determine the purity of the isolated RNA, while the RNA integrity was assessed by capillary electrophoresis with the Agilent Bioanalyser 2100 (Agilent Technologies, USA).

Total RNA was reverse transcribed to cDNA using the RT^2^ First Strand Kit (Qiagen, Germany). Rat Insulin Signaling Pathway RT^2^ Profiler PCR Arrays (Qiagen, Germany) were used to analyze the differential gene expression. The cDNA was added to the RT^2^ SYBR Green qPCR Master Mix then aliquoted in the wells of the array plate. An ABI 7500 Instrument (ThermoFisher Scientific, USA) was used to perform the RT-qPCR, and relative gene expression was determined using the ΔΔCt method. Each array had 84 assay genes with 5 housekeeping genes (viz. *Actb, B2m, Hprt1, Ldha and Rplp1*) (**Table S2**), and a reverse transcription efficiency and DNA contamination control. Three RNA biological replicates were pooled and repeated, in triplicate, for each experimental condition.

The CT values were tabulated for data analysis on http://www.qiagen.com/geneglobe with samples assigned to control and test groups. The data were normalized to the panel of reference genes. Fold change/regulation was calculated with the ΔΔCt method: ΔCT was calculated between the gene of interest and average of reference genes, followed by ΔΔCT calculations [ΔCT (test group)- ΔCT (control group)]. Fold change was then calculated using the 2^ (-ΔCT) formula.

### Statistical Analysis

For image analysis, the Kruskal Wallis test was done (overall comparison of the four groups), by sex for each group, and a linear regression model (analyzed log-transformed data) followed by pairwise comparison for sex independently. The analysis of data was executed *via* Stat v13.a (StataCorp LP, USA). For ELISA, the mean and SEM and group comparisons were analyzed by row analysis, and Bonferroni tests with Graph Pad Prism 8 software (GraphPad software, USA). For gene expression, fold change (2^ (- Delta CT)) represented the normalized gene expression (2^ (- Delta CT)) in the test sample divided by the normalized gene expression (2^ (- Delta CT)) in the control sample. Fold regulation represented fold changes that were biologically significant: where fold regulation = 1 reflected no change, fold changes >1 indicated upregulation, and fold changes <1 indicated downregulation, with fold changes ≥1.5 and ≤1.5 reported as upregulated or downregulated genes, respectively. The p values were calculated with the Student’s t-test of the replicate 2^ (- Delta CT) values for each gene in the control group and 40F groups. For the statistical analysis, p < 0.05 was significant.

## Results

### Immunoreactivity for Insulin Receptor, Glut4 and FoxO1

#### Insulin Receptor Immunoreactivity

**Figure 1:**
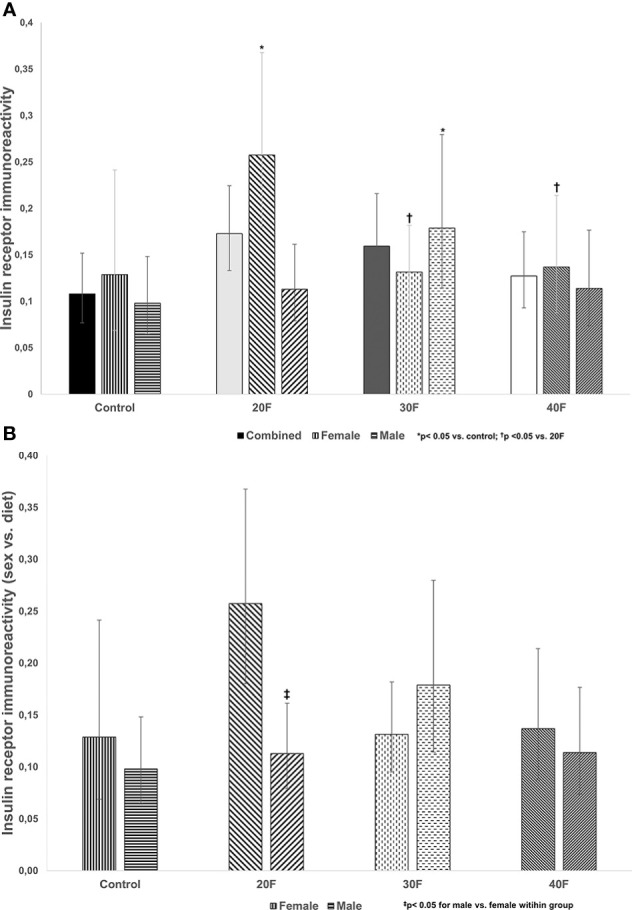
**(A)** Insulin receptor immunoreactivity. Combined neonates, female neonates, and male neonates maintained on either a control, 20% fat (20F), 30% fat (30F) or 40% fat (40F; high fat) diet. *p < 0.05 for 20F females compared to control females; and 30F males compared to control males. ^†^p < 0.05 for 30F and 40F females compared to 20F females. **(B)** Insulin receptor immunoreactivity (sex vs. diet). Female and male neonates maintained on either a control, 20% fat (20F), 30% fat (30F) or 40% fat (40F; high fat) diet. ^‡^p < 0.05 for 20F males compared to 20F females.

There were no differences in insulin receptor immunoreactivity in the combined phenotype (**Figure 1A**). However, 20F female neonates and 30F male neonates had increased insulin receptor immunoreactivity (**Figure 1A**). Further, 30F and 40F female neonates had reduced insulin receptor immunoreactivity compared to 20F female neonates (**Figure 1A**).

When comparing sex, 20F male neonates had reduced insulin receptor immunoreactivity relative to 20F female neonates (**Figure 1B**).

**Figure 2:**
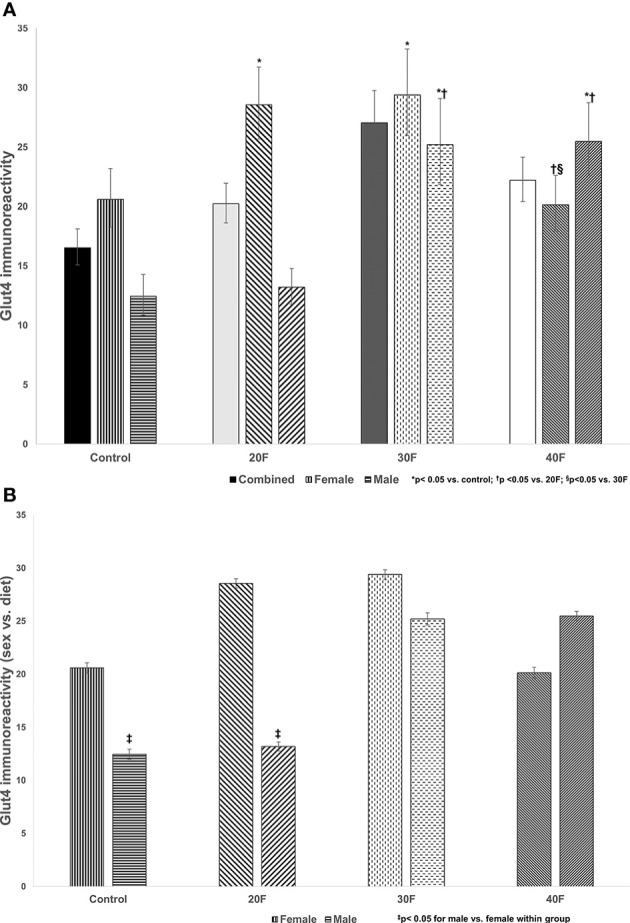
Glut4 immunoreactivity. Combined neonates, female neonates, and male neonates maintained on either a control, 20% fat (20F), 30% fat (30F) or 40% fat (40F; high fat) diet. *p < 0.05 for 20F and 30F females compared to control females; and 30F and 40F males compared to control males. ^†^p < 0.05 for 40F females compared to 20F females; and for 30F and 40F males compared to 20F males; ^§^p < 0.05 in 40F females compared to 30F females. **(B)** Glut4 immunoreactivity (sex vs. diet). Female and male neonates maintained on either a control, 20% fat (20F), 30% fat (30F) or 40% fat (40F; high fat) diet. ^‡^p < 0.05 for control and 20F males compared to control and 20F females, respectively.

#### Glut4 Immunoreactivity

There were no differences in Glut4 immunoreactivity in the combined phenotype (**Figure 2A**). In female neonates, 20F and 30F females showed higher Glut4 immunoreactivity compared to the control and 40F females. However, in male neonates, 30F and 40F males showed an increase in Glut4 immunoreactivity compared to the control and 20F males.

**Figure 3:**
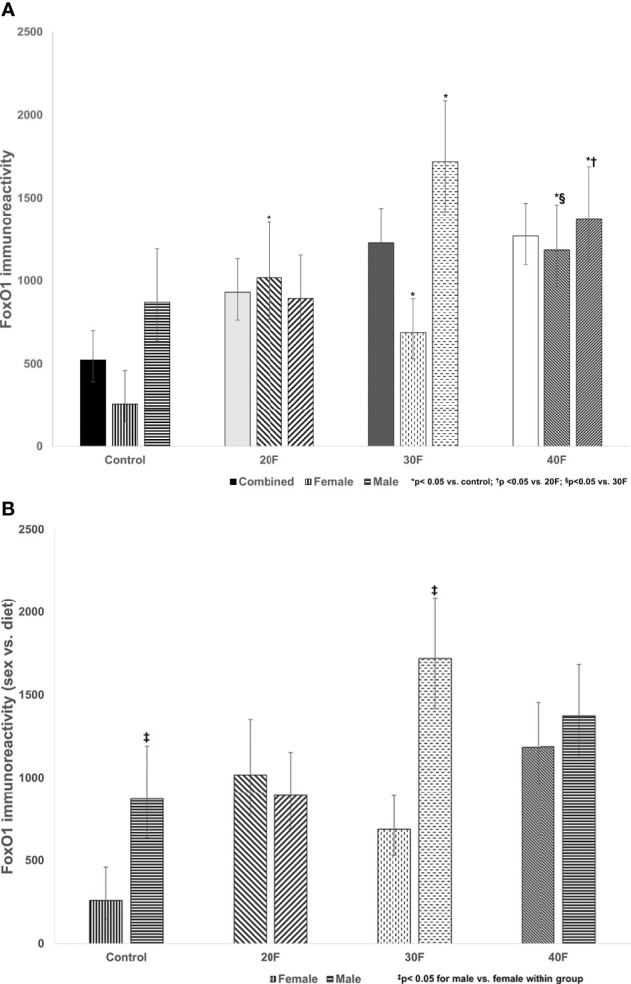
**(A)** FoxO1 immunoreactivity. Combined neonates, female neonates, and male neonates maintained on either a control, 20% fat (20F), 30% fat (30F) or 40% fat (40F; high fat) diet. *p < 0.05 for 20F, 30F and 40F females compared to control females; and 30F and 40F males compared to control males. ^†^p < 0.05 for 40F males compared to 20F males. ^§^p < 0.05 for 40F females compared to 30F females. **(B)** FoxO1 immunoreactivity (sex vs. diet). Female and male neonates maintained on either a control, 20% fat (20F), 30% fat (30F) or 40% fat (40F; high fat) diet. ^‡^p < 0.05 for control and 30F males compared to control and 30F females, respectively.

When comparing sex, control and 20F males had reduced Glut4 immunoreactivity compared to control and 20F females respectively (**Figure 2B**).

#### FoxO1 Immunoreactivity

There were no differences in FoxO1 immunoreactivity in the combined phenotype (despite what appeared to be increases in 20F, 30F and 40F neonates, that were non-significant) (**Figure 3A**). In female neonates, 20F, 30F and 40F females had higher FoxO1 immunoreactivity compared to the control; with 40F females also exhibiting higher FoxO1 immunoreactivity compared to 30F females (**Figure 3A**). In male neonates, 30F and 40F males had increased FoxO1 immunoreactivity compared to the control (similar for Glut4 immunoreactivity); and 40F males also had increased FoxO1 immunoreactivity compared to 20F males (**Figure 3A**).

When comparing sex, control and 30F male neonates had increased FoxO1 immunoreactivity relative to control and 30F female neonates respectively (**Figure 3B**).

### Glut4 Concentrations

Glut4 concentrations were reduced in 40F female neonates compared to 30F female neonates (**Table S1**). No samples were available for determining ELISA concentrations in 20F male offspring.

### Insulin Signaling Gene Expression

Eighty four genes involved in insulin signaling were investigated, with differential gene expression in high fat programmed female and male neonates (**Table S2**). By fold change, high fat programming altered 31 (37%) of the 84 genes of the insulin signaling pathway in both female and male neonates, of which 17 (20%) genes were unique, 7 (8%) were shared in female and male neonates, which affected 15 (18%) of the genes in female neonates and 16 (19%) of the genes in male neonates (**Tables 1**, **2** and **S2**).

In 40F female neonates, *Cebpa*, was upregulated 2.51 fold (p < 0.05), *Aebp1* (p = 0.098) and *Cfd* (p = 0.074) tended to be upregulated, whereas *Fbp*, *G6pc*, *Gck*, *Igfbp*, *Lep, Prkcz*, *Retn*, *Serpine 1* and *Ucp 1* had >1.5 fold increases (non-significant) (**Table 1**). In 40F female neonates, *Cebpb*, *Prkcg* and *Tg* were downregulated, with >1.5 fold decreases (non-significant) (**Table 1**).

**Table 1:** Differential gene expression in 40F female neonates.

	Gene	Fold regulation	P value
1	*Aebp1*	1.88	0.098
2	*Cebpa*	2.51	0.025*
3	*Cfd*	1.89	0.074
4	*Fbp1*	2.94	0.362
5	*G6pc*	1.60	0.423
6	*Gck*	3.12	0.249
7	*Igfbp*	2.02	0.341
8	*Lep*	2.02	0.164
9	*Prkcz*	1.67	0.146
10	*Retn*	2.77	0.346
11	*Serpine 1*	1.61	0.164
12	*Ucp 1*	18.19	0.352
13	*Cebpb*	-1.52	0.310
14	*Prkcg*	-1.75	0.210
15	*Tg*	-1.6	0.203

In 40F male neonates, *Adra1d* (p = 0.057) and *Prkcg* (p = 0.071) tended to be upregulated whereas *Cfd*, *Dok2*, *Fbp1*, *Frs3*, *G6pc*, *Igfbp1*, *Pklr*, *Retn*, *Srebf1* and *Ucp1* had >1.5 fold increases (non-significant) (**Table 2**). In 40F male neonates, *Hk2*, *Ins2*, *Kras* and *Pdpk1* had >1.5 fold decreases (non-significant) (**Table 2**).

**Table 2:** Differential gene expression in 40F male neonates.

	Gene	Fold regulation	P value
1	*Adra1d*	2.12	0.057
2	*Cfd*	1.98	0.265
3	*Dok2*	1.64	0.105
4	*Fbp1*	4.47	0.393
5	*Frs3*	1.56	0.298
6	*G6pc*	5.21	0.380
7	*Igfbp1*	3.59	0.369
8	*Pklr*	2.14	0.390
9	*Prkcg*	1.77	0.071
10	*Retn*	2.69	0.383
11	*Srebf1*	2.39	0.351
12	*Ucp1*	9.83	0.370
13	*Hk2*	-1.59	0.543
14	*Ins2*	-1.83	0.525
15	*Kras*	-1.55	0.299
16	*Pdpk1*	-1.69	0.549

## Discussion

Programming-induced alterations in insulin signaling is a consequence of maternal nutrition (e.g. over and undernutrition) during pregnancy (59) which influences offspring growth, development and health. Any alterations in nutrition during fetal life shape health outcomes that may present as early as neonatal life, and is expected to either be transient (e.g. corrected to some extent by eating healthier) or exacerbate and persist with consistent unhealthy nutrition and/or overnutrition. This reflects the metabolic agility and compensatory responses to fetal programming.

The control diet represented a low fat diet with 10% fat, with increasing fat content from 20%-40% in the experimental diets. The first part of the study reported on the varying fat content i.e. 20%, 30% and 40% fat, whereas the second part of the study focused on a high (40%) fat diet (i.e. high fat programming). Various diets can induce obesity and programming such as diets high in saturated fat, sucrose and calories. Westernized diets contain 36%–40% fat by energy (60). A 60% fat rodent diet diverges too greatly from control rodent diets, therefore rodent diets of 40%-45% fat more closely mimic diets consumed by people (60). Further, in rodent models of obesity, HFDs with high saturated fat content, more closely mimic human pathophysiology as they are often highly palatable and calorically dense to promote weight and fat gain (61). However, rodents have variable responses to diets, with sex, age and strain influencing their responses, and younger rodents and males may be more sensitive to obesity and its co-morbidities (61). In rodent models of developmental programming, the windows to induce obesity are short in duration, i.e. primarily the fetal and lactation phases, which may be insufficient to induce obesity with a HFD, unless the mothers are obese and/or diabetic or there are other obesogenic factors and/or compromised metabolic states to drive the obese phenotype.

### Varying Fat Content Alters Insulin Receptor, Glut4 and FoxO1 Immunoreactivity

The first phase of the study reported on altered immunoreactivity, in the neonatal heart, of the insulin receptor, Glut4 and FoxO1, factors with roles in maintaining cardiac physiology and insulin signaling, and demonstrated sex-specific effects after maintenance on diets varying in fat content i.e. 20%, 30% and 40% fat diets. One of the earliest defects in insulin resistant rats, due to HFDs, is dysfunctional auto-phosphorylation of the insulin receptor (62). Female neonatal offspring maintained, *in utero*, on a 20% fat diet and their male counterparts on a 30% fat diet, had increased insulin receptor immunoreactivity that may reflect a compensatory mechanism in response to their respective diets. In the combined phenotype, neonates maintained on 20% or 30% fat diets had higher body weights (56) with elevated glycemia in the former (55), which with the increased insulin receptor immunoreactivity reflect early events in the pathogenesis insulin resistance. High fat programmed neonates (combined phenotype) were hyperglycemic (63) with impaired insulin release from islets (64) and insulin resistant (65). Interestingly, in both female and male neonates maintained on a HFD, cardiac insulin receptor gene expression (data not shown) and immunoreactivity were unaltered.

A 30% fat diet (*in utero*) consistently programmed increased cardiac Glut4 immunoreactivity in both female and male neonates. Cardiac Glut4 immunoreactivity was also increased in female neonates, after maintenance on a 20% fat diet and in males maintained on a HFD *in utero*, which again reflected a variation in the percentage of fat content to elicit an effect, which was lower in female neonates at 20%, and distinctive sexual dimorphic programming responses in cardiac Glut4 immunoreactivity. When comparing the diets per sex, the control and 20F female neonates had higher cardiac Glut4 immunoreactivity relative to their male counterparts which demonstrated the sex-specific effects. Glut4 concentrations were not fully determined due to insufficient sampling, but in 40F female neonates, the Glut4 concentrations were reduced relative to the 30F neonates in alignment with immunoreactivity data.

FoxO1 is implicated in the pathogenesis of diabetic cardiomyopathy, and has a role in the regulation of dysfunctionalities in cardiac glucose and fatty acid metabolism (66–68). In female neonates, cardiac immunoreactivity for FoxO1 was consistently increased after maintenance on either a 20%, 30% or 40% fat diet. Thus, there was a lower fat threshold to elicit a consistent programming response in female neonates, and the increased immunoreactivity for FoxO1 may be an adaptive programming response that was consistent irrespective of varying dietary fat content. In male neonates maintained on a 30% or 40% fat diet, cardiac immunoreactivity for FoxO1 was increased reflecting a clear programming response at a higher fat threshold, with no programming effect with a 20% fat diet. These changes mirrored the cardiac Glut4 immunoreactivity which demonstrated consistent programming effects in male neonates in FoxO1 and Glut4 immunoreactivity. Unlike insulin receptor and Glut4, the cardiac immunoreactivity for FoxO1 was reduced in control and 30F female neonates compared to male neonates, which again revealed distinctive sex-specific programming effects.

In the combined neonatal data, there were no differences in cardiac insulin receptor, Glut4 or FoxO1 immunoreactivity. However, when accounting for sex, differences were apparent, thus highlighting the importance of sex-specific studies and effects. There is variable capacity for insulin sensitivity in females and males (69, 70), with sexual dimorphic effects of fetal cardiac programming due to an unfavorable *in utero* environment (49) (e.g. high fat programming or protein deficiency *in utero*) that trigger sex-specific metabolic derangements in offspring (51). In female neonates, maintenance on a 20% fat diet revealed a consistent increase in insulin receptor, Glut4 and FoxO1 immunoreactivity that was mirrored in male neonates maintained on a 30% fat diet. Thus there were (i) distinctive sex-specific effects on cardiac insulin signaling and (ii) lower dietary fat content induced changes in female neonates.

### High Fat Programming Alters Cardiac Insulin Signaling

Maternal nutrition, during gestation and/or lactation, largely determines fetal and neonatal growth and development and shapes offspring’s health outcomes. A maternal HFD during gestation diminishes maternal metabolism and physiology and, through high fat programming, provides a suboptimal intrauterine environment for fetal growth and development, thereby conferring unfavorable cardiac outcomes to offspring (71). As a cardiac stressor, high fat programming alters the expression of cardiac factors that modify cardiac structure and function (71), i.e. may induce cardiac remodeling, with specific programming induced alterations reported across species [reviewed in (71)]. In high fat programmed neonates (combined phenotype), heart weights were reduced (55) suggesting some heart stunting and possible structural compromization and/or modifications. Insulin signaling gene expression was studied in high fat programmed female and male neonates (second phase of the study). This is the first study, to our knowledge, to report on the upregulation of cardiac *Cebpa* mRNA expression in high fat programmed female neonatal offspring. High fat programmed female offspring showed a 2.5 fold increase in *Cebpa* mRNA expression. Cebpa, which is required for terminal adipocyte differentiation, is activated in the epicardium due to developmental cues and stress signals (72), and has roles in regulating postnatal systemic energy homeostasis and lipid storage. In embryogenesis, mitogenic factors are secreted by the epicardium for cardiomyocyte proliferation and for multipotent progenitor cells to develop the heart’s vasculature and fibrous architecture (73). Interestingly, in female neonates, *Cebpa* was upregulated but *Cebpb* was down regulated. Mice with reduced cardiac C/EBPβ (Cebpb) levels displayed resistance to cardiac failure after pressure overload (74). In high fat programmed female neonates, *Cebpa* upregulation may be triggered in response to stress (72) whereas *Cebpb* downregulation may be an adaptive cardioprotective mechanism (74), However, early molecular responses and adaptations need to be supported with protein studies (as transcripts may not necessarily be translated i.e. mRNA and protein expression may not correlate for a specific factor) and contextualized with cardiac physiological data. These limitations can be addressed in future studies.

The different insulin signaling genes that tended to significance and some genes that expressed fold changes ≥1.5 are discussed to discern their potential interrelations and roles in cardiovascular disease, diabetes and obesity. The overexpression of adipocyte enhancer-binding protein 1 (Aebp1) promoted atherosclerosis in AEBP1-transgenic mice with hyperlipidemia and atherosclerotic lesions in their proximal aortas (75). In obese individuals, elevated leptin may contribute to low-grade inflammation, rendering them more susceptible to cardiovascular diseases; whereas in dilated cardiomyopathy, leptin is a biomarker for the progression of heart failure independent of immune responses (76). *Aebp1* (p = 0.098) tended to be upregulated 1.9 fold similar to a 2 fold increase (non-significant) in leptin (*Lep*) expression, which could suggest cardiac inflammation and injury. Complement factor D (*Cfd* or adipsin), a serine protease, has a role in the activation of the alternative pathway of the complement system (77, 78). In female neonates, *Cfd* tended to be upregulated (there was also a non-significant increase in male neonates). In adipose tissue, adipsin mRNA abundance was increased during fasting in normal rats and in insulin deficient diabetes induced by streptozotocin (79). Further, in humans, plasma adipsin was elevated in obesity (80–82) and in coronary artery disease (83). The upregulation of *Cfd* in female and male neonates may also reflect a cardiac adaptive mechanism.

Adrenoceptor alpha 1D (Adra1D) is the predominant subtype in human coronary arteries (84). The α1-adrenergic receptor is expressed in the myocardium and vasculature of humans and rodents, regulates cardiovascular physiology, and was recently reported to be cardioprotective e.g. involved in hypertrophy, ischemic pre-conditioning and protection from apoptosis (85, 86). Thus in high fat programmed male neonates, the upregulation of *Adra1D* may be linked to cardioprotection (85, 86).

Although other protein kinase C (PKC) isoforms may have more prominent roles in cardiovascular physiology, *Prkcg* is involved in blood pressure modulation at the central nervous system level (87) and may protect neural tissue from ischemia. The PKCγ (Prkcg) isoform is expressed mainly in cells in the brain, neuronal tissues, the lens and retina. PKCα, β, δ and ϵ are expressed in the heart with PKCϵ playing a protective role (88). PKCϵ is involved in cardiac preconditioning (89). The brain and eye contain PKCγ and PKCϵ which may protect against stroke and neural ischemia (90). The C1 domains in PKCγ and PKCϵ are open and easily activated that enable their activation by oxidative signals and reactive oxygen species (ROS) (88). Both PKCγ and PKCϵ have roles in controlling the mitochondria and gap junctions during ischemic stress in neural tissues and the heart, respectively (88). *Prkcg* may play a role in the communication between cells and contribute to the positive inotropic effect induced by the α-adrenergic receptors (91, 92) which establishes a link between *Adra1d* and *Prkcg*. In the male offspring maintained on a HFD, the expression of *Adra1d* (p = 0.057) and *Prkcg* (p = 0.071) were increased 2 fold and 1.8 fold respectively, that any reflect a protective and/or regulatory response by these two genes, in the neonatal male heart after high fat programming.

Several genes in the insulin signaling pathway displayed >1.5 fold increases, albeit non-significant, in both female and male neonates viz. *Cfd* (already discussed), *Fbp1*, *G6pc*, *Igfbp* and *Retn* reflecting similar effects in both sexes. However, this was specific to the insulin signaling factor as *Pkrg* had a fold decrease in female neonates but a fold increase in male neonates, and there were also several unique genes with >1.5 fold changes per sex. Further investigation with additional pathway arrays and the determination of maternal and paternal gene expression profiles may provide further insights.

Fbp1 (or Folbp1) transports folate and appears to modulate glycerol gluconeogenesis in the liver, plays a role in embryogenesis in mice (93) and may regulate appetite and adiposity. Fbp1 may also mediate the transferring of maternal folate to embryos during neurulation (93) and susceptibility to heart defects (94). Fbp1 gene expression was decreased in the offspring of diabetic rats and in embryos cultured in high glucose (30 mmol/l glucose) after 24 hours of culture (95). G6PC is a key enzyme in glucose homeostasis. The impact of the fold increases in *Fbp1* and *G6pc* in the neonatal heart remain undefined.

IGFBP1 promotes neovascularization in response to ischemia, is required for the endothelium to respond appropriately to injury (96) and may be implicated in obesity. Retn (Resistin) induces insulin resistance in rodents, plays a role in atherosclerosis and cardiovascular disease, induces nuclear factor kappa-light-chain-enhancer of activated B cells (NFκB) activity and activates MAPKs such as Erk or p38 and Akt (97). Further, there are high resistin levels in cardiomyocytes derived from type 2 diabetic hearts, with resistin overexpression altering cardiac contractility and promoting cardiac hypertrophy potentially through the IRS1/MAPK pathway (98). In humans, circulating resistin correlated with inflammatory markers and predicted coronary atherosclerosis to connect metabolic signals, inflammation and atherosclerosis (99) and was linked to obesity-induced inflammation and cardiovascular events (100), evident by correlations with proinflammatory cytokines, lipids and systolic and diastolic blood pressure in obese adolescents with metabolic syndrome (100). The fold increases of *Igfbp* and *Retn* support a response to cardiac inflammation, stress and/or injury, in alignment with upregulation of *Cebpa*, and fold increases of *Aebp1*, *Cfd* (adipsin), *Adra1d* and *Prkcg*. Further, there was high upregulation in *Ucp1* mRNA in both sexes (an 18 fold increase in females and a 9.8 fold increase in males). UCP1 activity is induced during ischemia-reperfusion and mitigates reperfusion-induced damage, likely through lowering mitochondrial hyperpolarization at reperfusion, and reducing ROS production (101). Increased *Ucp1* mRNA expression, in high fat programmed female and male offspring, may be suggestive of heart injury and inflammation. Further investigation is required by conducting an inflammatory array in cardiac tissue and to further elucidate pathways affected by high fat programming, that may become more impactful over the offspring’s life-course.

### Sex-Specific Differences and Cardiovascular Disease

In the neonates maintained on diets varying in fat content, including high fat programmed neonates, there were distinctive sex-specific differences in the expression profiles of cardiac and insulin signaling factors. In the combined phenotype, differences in expression of factors were undetected. Sex is a determinant of alterations in cardiac physiology and structure (102) evident, in humans and rodents, by sex differences in arterial blood pressure (103–105). A maternal HFD induced cardiac hypertrophy; in adult male rat offspring, it increased cardiac susceptibility to ischemic-reperfusion injury, and differentially regulated cardiac angiotensin II (AngII), AngII receptor type 1 (AGTR1) and type 2 (AGTR2) expression through various mechanisms involved in the sex-specific alterations (49). Female db/db mice gained weight and were hypertensive with greater increases in left ventricular mass and worse diastolic dysfunction, whereas male db/db mice had accelerated microvascular rarefaction (102), highlighting the sex-specific cardiomyopathy associated with metabolic disease mirroring human diabetes, obesity and metabolic dysfunction (102). Female spontaneously hypertensive rats had delayed aortic dysfunction and associated myocardial remodeling that was dependent on sex-specific differences in levels of local angiotensin type 2 receptor (AT_2_R) and Mas receptor (MasR) (106). Further, they better preserved aortic endothelial function, had lower activities of matrix metalloproteinase 2 (MMP2), preserved elastin, had less fibrosis, and developed less left ventricular hypertrophy and cardiac fibrosis (106). These studies reveal sex-specific effects in cardiovascular disease, different cardiac pathologies in females and males, and some delays in the onset of cardiovascular disease (possibly cardioprotection) in females, in support of some of our findings.

## Conclusion

Maintenance on diets varying in fat content during fetal life altered the expression of cardiac factors and induced sex-specific changes. A 20% fat diet in female neonates and a 30% fat diet in male neonates were sufficient to induce differences, evident by increased immunoreactivity for insulin receptor, Glut4 and FoxO1. In female neonates maintained on a 40% fat diet (high fat programmed), *Cebpa* mRNA expression was upregulated that may reflect cardiac stress. In high fat programmed offspring, the fold increases in other insulin signaling genes viz. *Aebp1*, *Cfd* (adipsin), *Adra1d*, *Prkcg*, *Igfbp*, *Retn* (resistin) and *Ucp1* may suggest an adaptative response to cardiac inflammation, stress and/or injury. Programming with varying fat content altered cardiac factor immunoreactivity in neonatal offspring that were sex-specific. High fat programming also altered insulin signaling gene expression in a sex-specific manner. Therefore, diet and sex are determinants of cardiac physiology and pathophysiology, that reflect divergent mechanisms in female and male neonatal offspring.

## Data Availability Statement

Additional data generated from the current study are available from the corresponding author upon request.

## Ethics Statement

The animal study was reviewed and approved by South African Medical Research Council.

## Author Contributions

AG conducted the immunoreactivity, image analysis and ELISA experiments, and helped to draft the manuscript. SG conducted the gene expression studies and helped to draft the manuscript. MC designed the study, drafted and reviewed the manuscript. All authors contributed to the article and approved the submitted version.

## Funding

This study was funded by the South African Medical Research Council (SAMRC) under the Masters/PhD Internship scholarship programme, the College of Health Sciences, University of KwaZulu-Natal (UKZN) and the National Research Foundation (South Africa).

## Conflict of Interest

The authors declare that the research was conducted in the absence of any commercial or financial relationships that could be construed as a potential conflict of interest.

## Publisher’s Note

All claims expressed in this article are solely those of the authors and do not necessarily represent those of their affiliated organizations, or those of the publisher, the editors and the reviewers. Any product that may be evaluated in this article, or claim that may be made by its manufacturer, is not guaranteed or endorsed by the publisher.

## Supplementary Material

Supplementary Table 1 Glut4 concentrations (ng/ml).

Supplementary Table 2 Genes assessed.
