# Supplementary material for: Programming With Varying Dietary Fat Content Alters Cardiac Insulin Receptor, Glut4 and FoxO1 Immunoreactivity in Neonatal Rats, Whereas High Fat Programming Alters Cebpa Gene Expression in Neonatal Female Rats
Source: Front Endocrinol (Lausanne). 2022 Jan 5;12:772095. doi: 10.3389/fendo.2021.772095 (PMC8766637; doi:10.3389/fendo.2021.772095)
Supplement: Supplementary Table 1 — Glut4 concentrations (ng/ml). [file Table_1.docx]

**Table S1. Glut4 concentrations (ng/ml)**

|  | **Control** | **20F** | **30F** | **40F** |
| --- | --- | --- | --- | --- |
| Combined | 0.27 ± 0.25 | - | 0.38 ± 0.27 | 0.12 ± 0.05 |
| Female | 0.14 ± 0.05 | 0.18 ± 0.13 | 0.52 ± 0.25 | 0.11 ± 0.07^§^ |
| Male | 0.40 ± 0.31 | - | 0.25 ± 0.27 | 0.13 ± 0.05 |

^§^p < 0.05 compared to 30F neonates
