# Supplementary material for: Programming With Varying Dietary Fat Content Alters Cardiac Insulin Receptor, Glut4 and FoxO1 Immunoreactivity in Neonatal Rats, Whereas High Fat Programming Alters Cebpa Gene Expression in Neonatal Female Rats
Source: Front Endocrinol (Lausanne). 2022 Jan 5;12:772095. doi: 10.3389/fendo.2021.772095 (PMC8766637; doi:10.3389/fendo.2021.772095)
Supplement: Supplementary Table 2 — Genes assessed. [file Table_2.docx]

**Table S2. Genes assessed**

|  | **All genes** | **Unique genes** | **Shared genes** |
| --- | --- | --- | --- |
| 1 | *Acaca* | *Adra1d* | *Cfd* |
| 2 | *Acox1* | *Aebp1* | *Fbp1* |
| 3 | *Adra1d* | *Cebpa* | *G6pc* |
| 4 | *Aebp1* | *Cebpb* | *Igfbp1* |
| 5 | *Akt1* | *Dok2* | *Prkcg* |
| 6 | *Akt2* | *Frs3* | *Retn* |
| 7 | *Akt3* | *Gck* | *Ucp1* |
| 8 | *Araf* | *Hk2* |  |
| 9 | *Bcl2l1* | *Ins2* |  |
| 10 | *Braf* | *Kras* |  |
| 11 | *Cap1* | *Lep* |  |
| 12 | *Cbl* | *Pdpk1* |  |
| 13 | *Cebpa* | *Pklr* |  |
| 14 | *Cebpb* | *Prkcz* |  |
| 15 | *Cfd* | *Serpine 1* |  |
| 16 | *Dok1* | *Srebf1* |  |
| 17 | *Dok2* | *Tg* |  |
| 18 | *Dok3* |  |  |
| 19 | *Dusp14* |  |  |
| 20 | *Eif2b1* |  |  |
| 21 | *Eif4ebp1* |  |  |
| 22 | *Ercc1* |  |  |
| 23 | *Fasn* |  |  |
| 24 | *Fbp1* |  |  |
| 25 | *Fos* |  |  |
| 26 | *Frs2* |  |  |
| 27 | *Frs3* |  |  |
| 28 | *G6pc* |  |  |
| 29 | *Gab1* |  |  |
| 30 | *Gcg* |  |  |
| 31 | *Gck* |  |  |
| 32 | *Gpd1* |  |  |
| 33 | *Grb10* |  |  |
| 34 | *Grb2* |  |  |
| 35 | *Gsk3b* |  |  |
| 36 | *Hk2* |  |  |
| 37 | *Hras* |  |  |
| 38 | *Igf1r* |  |  |
| 39 | *Igf2* |  |  |
| 40 | *Igfbp1* |  |  |
| 41 | *Ins1* |  |  |
| 42 | *Ins2* |  |  |
| 43 | *Insl3* |  |  |
| 44 | *Insr* |  |  |
| 45 | *Irs1* |  |  |
| 46 | *Irs2* |  |  |
| 47 | *Jun* |  |  |
| 48 | *Klf10* |  |  |
| 49 | *Kras* |  |  |
| 50 | *Ldlr* |  |  |
| 51 | *Lep* |  |  |
| 52 | *Map2k1* |  |  |
| 53 | *Mapk1* |  |  |
| 54 | *Mtor* |  |  |
| 55 | *Nos2* |  |  |
| 56 | *Npy* |  |  |
| 57 | *Pck2* |  |  |
| 58 | *Pdpk1* |  |  |
| 59 | *Pik3ca* |  |  |
| 60 | *Pik3cb* |  |  |
| 61 | *Pik3r1* |  |  |
| 62 | *Pik3r2* |  |  |
| 63 | *Pklr* |  |  |
| 64 | *Pparg* |  |  |
| 65 | *Ppp1ca* |  |  |
| 66 | *Prkcg* |  |  |
| 67 | *Prkcz* |  |  |
| 68 | *Prl* |  |  |
| 69 | *Ptpn1* |  |  |
| 70 | *Raf1* |  |  |
| 71 | *Retn* |  |  |
| 72 | *Rps6ka1* |  |  |
| 73 | *Rras* |  |  |
| 74 | *Rras2* |  |  |
| 75 | *Serpine1* |  |  |
| 76 | *Shc1* |  |  |
| 77 | *Slc27a4* |  |  |
| 78 | *Slc2a1* |  |  |
| 79 | *Slc2a4* |  |  |
| 80 | *Sos1* |  |  |
| 81 | *Srebf1* |  |  |
| 82 | *Tg* |  |  |
| 83 | *Ucp1* |  |  |
| 84 | *Vegfa* |  |  |
| 85 | *Actb^•^* |  |  |
| 86 | *B2m^•^* |  |  |
| 87 | *Hprt1^•^* |  |  |
| 88 | *Ldha^•^* |  |  |
| 89 | *Rplp1** |  |  |

*^•^*housekeeping genes
